# Comparative Evaluation of Infected and Noninfected *Amblyomma triste* Ticks with *Rickettsia parkeri*, the Agent of an Emerging Rickettsiosis in the New World

**DOI:** 10.1155/2013/402737

**Published:** 2013-07-08

**Authors:** F. A. Nieri-Bastos, M. P. J. Szabó, R. C. Pacheco, J. F. Soares, H. S. Soares, J. Moraes-Filho, R. A. Dias, M. B. Labruna

**Affiliations:** ^1^Faculdade de Medicina Veterinária e Zootecnia, Universidade de São Paulo, Avenida Prof. Orlando Marques de Paiva 87, Cidade Universitária, 05508-270 São Paulo, SP, Brazil; ^2^Faculdade de Medicina Veterinária e Zootecnia, Universidade Federal de Uberlândia, Avenida Pará 1720, Campus Umuarama-Bloco 2T, 38400-902 Uberlândia, MG, Brazil; ^3^Faculdade de Agronomia e Medicina Veterinária, Universidade Federal do Mato Grosso, Avenida Fernando Corrêa da Costa 2367, Boa Esperança, 78060-900 Cuiabá, MT, Brazil

## Abstract

The distribution of *Rickettsia parkeri* in South America has been associated with *Amblyomma triste* ticks. The present study evaluated under laboratory conditions two colonies of *A. triste*: one started from engorged females that were naturally infected by *R. parkeri* (designated as infected group); the other started from noninfected females (designated as control group). Both colonies were reared in parallel for five consecutive generations. Tick-naïve domestic rabbits were used for feeding of each tick stage and generation. *R. parkeri* was preserved by transstadial maintenance and transovarial transmission in *A. triste* ticks for five consecutive generations, because all tested larvae, nymphs, and adults from the infected group were shown by PCR to contain rickettsial DNA. All rabbits infested by larvae, nymphs, and adults from the infected group seroconverted, indicating that these tick stages were all vector competent for *R. parkeri*. Expressive differences in mortality rates were observed between engorged nymphs from the infected and control groups, as indicated by 65.9% and 92.4% molting success, respectively. Our results indicate that *A. triste* can act as a natural reservoir for *R. parkeri*. However, due to deleterious effect caused by *R. parkeri* on engorged nymphs, amplifier vertebrate hosts might be necessary for natural long-term maintenance of *R. parkeri* in *A. triste*.

## 1. Introduction

The bacterium *Rickettsia parkeri *was firstly reported infecting the *Amblyomma maculatum* tick in Texas >70 years ago [1]. Although its pathogenicity for humans was suspected or speculated during the following decades [2], *R. parkeri* was recognized as a human tick-borne pathogen only in 2004 [3]. At that same year, *R. parkeri *was reported infecting *Amblyomma triste *ticks in Uruguay [4]. Few years later, *R. parkeri-*infected *A. triste *ticks were reported in Brazil [5] and Argentina [6]. More recently, *A. maculatum *ticks were found infected by *R. parkeri *in Peru [7]. While a number of human cases of *R. parkeri *rickettsiosis have been recently described in the United States, all associated with *A. maculatum *ticks [2, 8–10], in South America, human cases were reported only in Uruguay and Argentina, generally associated with *A. triste *ticks [11, 12]. In Brazil, human cases of a novel rickettsiosis caused by an agent closely related to *R. parkeri *have been reported [13, 14]; however, no cases of human infection with *R. parkeri *sensu stricto have been reported in the country, despite the presence of the agent infecting *A. triste *ticks [5]. 

Reported information has indicated that the distribution of *R. parkeri *sensu stricto has been generally associated with *A. maculatum *in North America and *A. triste *ticks in South America, where usually *≈*10% of field collected adult ticks have been found infected by *R. parkeri* [4–6, 10, 15]. All these tick reports, as well as the human clinical cases reported in the United States and Argentina, refer to rickettsial genotypes 100% identical to the Maculatum 20 strain of *R. parkeri, *which is the original strain first described by Parker in 1939. Therefore, it is possible that a single *R. parkeri *genotype (namely, Maculatum 20) circulates among *A. maculatum *and *A. triste *populations in the New World, where both tick species use medium- to large-sized mammals as hosts for adult ticks, and chiefly small mammals and ground feeding birds for immature tick stages [16–18]. Because our knowledge on the ecology of *R. parkeri *rickettsiosis is still very incipient, the present study evaluated for the first time (i) the occurrence of transovarial transmission (passage of viable rickettsiae from tick female to its offspring) and transstadial transmission (maintenance of the infection during tick ontogeny) of the agent in one of its natural vectors, (ii) if the infection with *R. parkeri *causes any deleterious effect on its tick host, and (iii) the vector competence of all feeding stages of *A. triste*.

## 2. Material and Methods

### 2.1. Formation of Tick Colonies

In September 1 2008, *A. triste *adult ticks (38 males, 19 females) were collected from a road-killed marsh deer (*Blastocerus dichotomus*) in the Km 177 of the BR 262 highway (20°24′32.3′′S; 53°12′27.0′′W), within Agua Clara Municipality, state of Mato Grosso do Sul, Brazil. Ticks were brought alive to the laboratory, where female engorgement was completed by feeding on a tick-naïve uninfected New Zealand white rabbit (*Oryctolagus cuniculus*). A total of 14 females completed engorgement and were allowed to oviposit inside an incubator adjusted to 25°C and 90% relative humidity. Small samples of eggs (1 pool of 20 eggs/female) and the subsequent hatched larvae (1 pool of 20 larvae/female) derived from each of the 14 engorged females were subjected to DNA extraction and subsequently tested by PCR using primers CS-78 and CS-323, which target a 401 bp fragment of the citrate synthase gene (*gltA*) of possibly all *Rickettsia *species [19]. Egg and larval samples from 4/14 (28.6%) engorged females yielded PCR products that after DNA sequencing, were shown to be 100% identical to *R. parkeri *previously reported in *A. triste *from Brazil, Uruguay, and Argentina [5, 6, 20]. Based on these results, the larval offspring (F_1_) from two infected females were pooled to start the infected group of the present study. At the same time, the larval offspring from two PCR-negative noninfected females were pooled to start the control group. 

### 2.2. Tick Infestations on Rabbits

The F_1_ unfed larvae of infected and control groups were reared separately in the laboratory for 5 consecutive generations until they were F_5_ unfed nymphs. Throughout the experiment, infestations with infected and control groups were done in parallel; all infested animals were held in the same room under the same environmental conditions. New Zealand white rabbits were infested with larvae, nymphs, and adult ticks, as previously described for *A. triste *[21]. In each infestation with a given tick stage from the infected or control group, a different tick-naïve rabbit was used. Larval and nymphal infestations consisted of *≈*3,000 and 500 ticks, respectively, per host, whereas adult infestations consisted of 10 to 25 couples per rabbit. Infestation of each animal was performed inside a feeding chamber glued to its shaved dorsum, as previously described [22]. All infested animals had their temperature rectally measured daily from the day of infestation (day 0) to 21 days after infestation. Rabbits were considered febrile if rectal temperatures were >40.0°C [23]. Naturally detached engorged larvae, nymphs, or female ticks were recovered daily from the feeding chambers of the infested animals of both groups and immediately taken to a single incubator adjusted to 25°C and 95% relative humidity for molting (for engorged larvae and nymphs) or for egg laying and incubation (for engorged females). Engorged females had their individual weight measured the day they detached from the host. In addition, the total egg mass deposited by each female was weighed on the day of the end of oviposition, and a conversion efficiency index (CEI = mg egg mass/mg engorged female × 100), which measures the efficiency with which a female tick converts body weight into eggs [24], was determined for each female that oviposited. Percentage of egg hatching for each egg mass was visually estimated [25]. 

### 2.3. Molecular Tests on Ticks

During the experiment, random samples of 30 unfed larvae and 20 unfed nymphs of each of the generations F_1_ through F_5_, and 10 unfed adults of generations F_1_–F_4_ from both infected and control groups, were individually submitted to DNA extraction by using guanidine thiocyanate, as previously described [26]. A higher number of larvae were processed because this was the most numerous active stage; at the same time, less adults were processed because this was the least numerous developmental state. Five microliters of each tick DNA template (approximately 500 ng of DNA) was used for PCR using the primers CS-78 (forward) 5′-GCA AGT ATC GGT GAG GAT GTA AT-3′ and CS-323 (reverse) 5′-GCT TCC TTA AAA TTC AAT AAA TCA GGA T-3′, which amplify a 401 bp fragment of the rickettsial *gltA *gene [19]. PCR conditions were as follows: DNA melting at 95°C for 15 sec., primer annealing 55°C for 30 sec., and polymerase extension at 72°C for 30 sec., for 35 cycles. For each set of reactions, negative (5 *μ*L of water) and positive (5 *μ*L of DNA extracted from *R. parkeri* strain NOD-infected cells) controls were included.

### 2.4. Serological Tests

All tick-infested rabbits were tested for seroconversion to *R. parkeri* antigens. For this purpose, blood samples were collected at 0 and 21 days after infestation; these samples were tested for anti-*R. parkeri* (strain At24) reactive antibodies by immunofluorescence assay (IFA), as previously described [27]. Briefly, sera were diluted in phosphate-buffered saline (PBS) and screened at a dilution of 1 : 64 on 12-well antigen slides. The slides were incubated, washed, then incubated with fluorescein isothiocyanate-labelled conjugate goat anti-rabbit immunoglobulin G (IgG) (Sigma Diagnostics, St. Luis, Mo, USA) and washed again, mounted with buffered glycerin, and read using an ultraviolet microscope (BX60; Olympus Corp., Tokyo, Japan) at 400x magnification. A rabbit serum previously identified as nonreactive (negative control) and a rabbit serum known to be reactive (positive control) were tested on each slide. Serum samples reacting at the screening dilution were tested in serial twofold dilutions to determine the endpoint titer.

### 2.5. Isolation of Rickettsiae from Ticks

Four F_3_ and four F_4_ unfed adult ticks were processed by the shell vial technique for isolation of rickettsiae in Vero cell culture as described by Marrero and Raoult [28] and modified by Labruna and others [19]. Briefly, each tick was triturated in sterile brain heart infusion broth and the resultant tick homogenate inoculated into shell vials containing a monolayer of confluent Vero cells. After inoculation, the shell vials were centrifuged for 1 h at 700 g at 22°C. Rickettsial infection was checked by Gimenez staining, and the rickettsial isolate was considered established after at least three passages in Vero cells, each reaching more than 90% of infected cells [19]. Third passage-infected cells were submitted to DNA extraction using the DNeasy tissue Kit (Qiagen, Chatsworth, CA) and tested by two PCR protocols: one using primers CS-78 and CS-323 targeting the rickettsial *gltA *gene, as described above for ticks, and another protocol using primers Rr190.70p and Rr190.701n, targeting a 631 bp fragment of the rickettsial 190 kDa outer membrane protein gene (*ompA*), as previously described [29]. PCR products were purified using ExoSAP-IT (USB Corp., Cleveland, OH, USA) and underwent DNA sequencing in an ABI automated sequencer (Applied Biosystems/Perkin Elmer, model ABI Prism 310 Genetic, Foster City, CA, USA), and the resultant sequences were compared with GenBank data by BLAST analysis (http://blast.ncbi.nlm.nih.gov/Blast.cgi).

### 2.6. Statistical Analysis

During the experiment, tick biologic parameters were compared between infected and control groups. For this purpose, F_5_ larval and F_1_–F_4_ nymphal molting success and F_1_–F_4_ female oviposition success (i.e., death of engorged ticks) were compared by the chi-square test. In addition, weight of engorged females and their corresponding egg masses, percentage of egg hatching, and CEI values were compared by Student *t* test. Values were considered significantly different when *P* < 0.05. The study was approved by the Bioethical Committee in Animal Research of the Faculty of Veterinary Medicine of the University of Sao Paulo.

## 3. Results

The infected tick group remained infected by rickettsiae through 5 consecutive generations, until the end of the experiment (F_5_ unfed nymphs). In all infestations performed with ticks from this group, rabbits seroconverted to *R. parkeri *antigens with endpoint titers varying from 256 to 1024 (Table 1). In contrast, no rabbit infested with ticks from the control group seroconverted; that is, they were nonreactive for *R. parkeri* (serum dilution 1 : 64) at both 0 and 21 days after infestation. All rabbits from both infected and control groups remained afebrile during the study period.

All PCRs performed individually on unfed ticks (larvae, nymphs, and adults) from five consecutive generations of the infected group resulted in amplicons compatible with *R. parkeri *(Table 1). In contrast, no tick specimen from the five generations of the control group yielded PCR amplicons. Among 8 infected group-adult ticks processed by the shell vial technique, rickettsiae were successfully isolated and established in cell culture from 2 F_3_ and 3 F_4_ ticks. Rickettsiae were also isolated from other 2 F_3_ ticks and another F_4_ adult tick; however, the isolates were lost due to fungal or extracellular bacterial contamination. PCR products from the 5 established isolates yielded partial fragments of the rickettsial *gltA *and *ompA *genes, which after DNA sequencing, were shown to be identical to each other and 100% identical to the corresponding sequences of *R. parkeri *from the United States and South America, available in Genbank (EF102236, U59732, U43802, EF102238, and JN664898). These five isolates, designated as Agua Clara 1–5, have been deposited in the Rickettsial Collection of the Faculty of Veterinary Medicine of the University of São Paulo. 

Significantly more control group-engorged nymphs successfully molted to adults than the engorged nymphs of the infected group (Table 2); that is, mortality rate for engorged nymphs of four consecutive generations was always higher in the infected group. The numbers of engorged females that successfully oviposited were statistically similar between infected and control groups through the four tick generations (Table 2). Molting success of engorged larvae to nymphs was quantified only in the fifth generation. In this case, 77.3% (153/198) and 71.7% (345/481) of the engorged larvae of the control and infected groups successfully molted to nymphs. These proportions were not statistically different (*P* = 0.164).

Reproductive data of engorged females were generally similar between control and infected groups (Table 3). The only exceptions were the % egg hatching of egg masses laid by F_1_ and F_2_ females, which were significantly higher in the control group. However, when % egg hatching of the four generations was pooled, there was no significant difference between the groups. 

## 4. Discussion

In the study reported here, *R. parkeri* was preserved by transstadial maintenance and transovarial transmission in *A. triste* ticks for 5 consecutive generations, because all tested larvae, nymphs, and adults from the infected group were shown by PCR to contain rickettsial DNA, from the first to the fifth generation. In addition, infestations of rabbits with larvae, nymphs, and adults from all generations confirmed that ticks of these 3 developmental stages were infected by *R. parkeri* because all infested rabbits seroconverted through IFA. These results also confirm that larvae, nymphs, and adults of* A. triste* ticks are competent vectors of *R. parkeri*. Our results strongly support clinical and epidemiologic data that have implicated the *A. triste* tick as the main vector of *R. parkeri* sensu stricto in South America [4, 6, 12, 30]. 

Because all individual larvae of the infected group yielded rickettsial DNA by PCR, 100% transovarial transmission rate (the proportion of infected females giving rise to at least 1 infected larva) and 100% filial infection rate (proportion of infected larvae obtained from an infected female) were observed. In contrast, no individual larva from the control group yielded rickettsial DNA by PCR. These results are in agreement with the larval infestations, which resulted in seroconversion in all rabbits infested by larvae from the infected group and with no seroconversion in animals infested with control group larvae. Although transovarial transmission of spotted fever rickettsiae in ticks seems to occur worldwide [31], such information has never been previously reported for *R. parkeri*. 

Expressive differences in mortality rates were observed between engorged nymphs from the infected and control groups, as indicated by 65.9% and 92.4% molting success, respectively. Overall, these rates mean that the mortality of *R. parkeri*-infected engorged nymphs was nearly 5 times higher than the mortality of uninfected nymphs. Similar to the present study, expressive mortality rates were reported for *Rickettsia rickettsii-*infected engorged nymphs of *Dermacentor andersoni, *and* Rickettsia conorii*-infected *Rhipicephalus sanguineus *nymphs, when compared with uninfected sibling ticks [32–34]. These authors also observed higher mortality of infected engorged larvae, albeit not as expressive as those of infected engorged nymphs. In the present study, a nonsignificant higher mortality of *R. parkeri-*infected engorged larvae was observed, when compared to noninfected engorged larvae of the fifth generation. Unfortunately, we did not quantify larval molting success for generations F_1_–F_4_, precluding an overall comparison of data from the five larval generations.

On the other hand, our results demonstrated that both *R. parkeri*-infected and uninfected engorged females had overall similar survivorship and reproductive performance. These results contrast previous studies that reported higher mortality and lower reproductive performance of *R. rickettsii*-infected engorged females of *Dermacentor variabilis*, *D. andersoni,* and *Amblyomma aureolatum* ticks, when compared with uninfected sibling ticks [35, 36]. Tick mortality is much more influential on the tick population when it occurs in engorged females; that is, although each dead egg, larva, or nymph is only less 1 subsequent larva, nymph, or adult, respectively, in the tick population, a dead engorged female represents thousands of eggs fewer in the following generation. Based on this assumption, Labruna and others [36] argued that the very low *R. rickettsii-*infection rates (<1%) usually reported among ticks in nature are related to the expressive mortality of the infected engorged females. On the other hand, the much higher *R. parkeri-*infection rates (usually around 10%) generally reported for *A. triste *under natural conditions [4–6] could be related to the deleterious effect of this rickettsia mainly on the nymphal rather than on the adult stage.

## 5. Conclusions

Ticks naturally infected by *R. parkeri *were reared for five consecutive generations in the laboratory. The infection by *R. parkeri *was successfully maintained in the tick population by transstadial maintenance and transovarial transmission. These results indicate that *R. parkeri *could be maintained by *A. triste *in nature over years; therefore, *A. triste *can act as a natural reservoir of *R. parkeri*. However, because of a notable deleterious effect caused by *R. parkeri* on engorged nymphs, infected ticks would disappear from the tick population in a long-term scenario, unless new lineages of infected ticks are created through horizontal transmission via amplifier vertebrate hosts, which remain unknown for *R. parkeri.*

## Figures and Tables

**Table 1:** Results of immunofluorescence assay (IFA) performed on rabbits infested with *Amblyomma triste* ticks from the infected group, and results of PCR targeting the rickettsial gene *gltA* performed on ticks of the infected group, through five consecutive generations (F_1_–F_5_) in the laboratory.

Tick stage and generation	IFA endpoint titer according to the tick infestation day		Number of PCR-positive ticks*/*Number of ticks tested by PCR (% infection)
	Day 0	Day 21	
Larva-F_1_	<64	1024	30/30 (100%)
Nymph-F_1_	<64	256	20/20 (100%)
Adult-F_1_	<64	512	10/10 (100%)
Larva-F_2_	<64	256	30/30 (100%)
Nymph-F_2_	<64	256	20/20 (100%)
Adult-F_2_	<64	256	10/10 (100%)
Larva-F_3_	<64	512	30/30 (100%)
Nymph-F_3_	<64	512	20/20 (100%)
Adult-F_3_	<64	1024	10/10 (100%)
Larva-F_4_	<64	256	30/30 (100%)
Nymph-F_4_	<64	1024	20/20 (100%)
Adult-F_4_	<64	256	10/10 (100%)
Larva-F_5_	<64	512	30/30 (100%)
Nymph-F_5_	<64	512	20/20 (100%)

**Table 2:** Molting and oviposition success of *Amblyomma triste* ticks infected by *Rickettsia parkeri* (infected group) and noninfected (control group) through four consecutive generations (F_1_ to F_4_) in the laboratory.

Tick generation	Number nymphs that molted to adults/Number recovered engorged nymphs (% molting success)		Number females that oviposited/Number recovered engorged females (% oviposition success)	
	Infected	Control	Infected	Control
F_1_	274/558 (49.1)	537/656 (81.9)*	18/19 (94.7%)	9/9 (100)
F_2_	1154/1580 (73.0)	1089/1093 (99.6)*	18/21 (85.7%)	7/7 (100)
F_3_	100/240 (41.7)	299/321 (93.1)*	10/10 (100)	7/7 (100)
F_4_	404/552 (73.2)	411/457 (89.9)*	10/10 (100)	5/5 (100)

Total	1932/2930 (65.9)	2336/2527 (92.4)*	56/60 (93.3)	28/28 (100)

*means that molting or oviposition success values for infected and control ticks (horizontal lines) of the same tick stage were significantly different (*P* < 0.05).

**Table 3:** Reproductive data of *Amblyomma triste* engorged females infected by *Rickettsia parkeri* (infected group) and noninfected (control group) through four consecutive generations (F_1_ to F_4_) in the laboratory.

Tick generation	Mean engorged weight (mg)^+^		Mean egg mass weight (mg)^+^		Mean CEI^+^		Mean % egg hatching^+^	
	Infected	Control	Infected	Control	Infected	Control	Infected	Control
F_1_	427.4 ± 145.5	479.2 ± 125.9	209.3 ± 89.0	231.4 ± 92.3	44.8 ± 11.5	45.0 ± 15.9	51.2 ± 29.8	72.2 ± 10.0*
F_2_	323.3 ± 131.0	328.3 ± 215.6	164.7 ± 68.2	168.7 ± 121.6	44.2 ± 11.7	48.5 ± 9.0	48.7 ± 27.4	73.6 ± 10.3*
F_3_	412.9 ± 89.7	303.1 ± 117.4	226.6 ± 63.4	158.0 ± 71.9	54.2 ± 4.4	50.0 ± 10.1	71.5 ± 11.8	73.6 ± 12.5
F_4_	407.0 ± 13.7	353.0 ± 11.3	218.7 ± 94.9	163.0 ± 85.6	51.6 ± 8.6	44.3 ± 13.1	71.0 ± 27.7	32.0 ± 37.5

Total	385.1 ± 136.1	375.0 ± 159.9	199.7 ± 81.5	185.1 ± 95.5	47.5 ± 10.7	47.0 ± 12.1	57.6 ± 27.6	65.7 ± 23.5

^+^values presented as mean ± standard deviation.

CEI: conversion efficiency index = egg mass weight/female engorged weight × 100.

*means significantly different (*P* < 0.05) values between infected and control groups (horizontal lines) for each tick biological parameter.
